# User Experience of COVID-19 Chatbots: Scoping Review

**DOI:** 10.2196/35903

**Published:** 2022-12-27

**Authors:** Becky K White, Annegret Martin, James Angus White

**Affiliations:** 1 School of Population Health Curtin University Perth Australia; 2 Reach Health Promotion Innovations Perth Australia

**Keywords:** COVID-19, chatbot, engagement, user experience, pandemic, global health, pandemic, digital health, health information

## Abstract

**Background:**

The COVID-19 pandemic has had global impacts and caused some health systems to experience substantial pressure. The need for accurate health information has been felt widely. Chatbots have great potential to reach people with authoritative information, and a number of chatbots have been quickly developed to disseminate information about COVID-19. However, little is known about user experiences of and perspectives on these tools.

**Objective:**

This study aimed to describe what is known about the user experience and user uptake of COVID-19 chatbots.

**Methods:**

A scoping review was carried out in June 2021 using keywords to cover the literature concerning chatbots, user engagement, and COVID-19. The search strategy included databases covering health, communication, marketing, and the COVID-19 pandemic specifically, including MEDLINE Ovid, Embase, CINAHL, ACM Digital Library, Emerald, and EBSCO. Studies that assessed the design, marketing, and user features of COVID-19 chatbots or those that explored user perspectives and experience were included. We excluded papers that were not related to COVID-19; did not include any reporting on user perspectives, experience, or the general use of chatbot features or marketing; or where a version was not available in English. The authors independently screened results for inclusion, using both backward and forward citation checking of the included papers. A thematic analysis was carried out with the included papers.

**Results:**

A total of 517 papers were sourced from the literature, and 10 were included in the final review. Our scoping review identified a number of factors impacting adoption and engagement including content, trust, digital ability, and acceptability. The papers included discussions about chatbots developed for COVID-19 screening and general COVID-19 information, as well as studies investigating user perceptions and opinions on COVID-19 chatbots.

**Conclusions:**

The COVID-19 pandemic presented a unique and specific challenge for digital health interventions. Design and implementation were required at a rapid speed as digital health service adoption accelerated globally. Chatbots for COVID-19 have been developed quickly as the pandemic has challenged health systems. There is a need for more comprehensive and routine reporting of factors impacting adoption and engagement. This paper has shown both the potential of chatbots to reach users in an emergency and the need to better understand how users engage and what they want.

## Introduction

### Background

On March 11, 2020, the World Health Organization (WHO) declared the COVID-19 outbreak a pandemic [1]. Since then, the virus has had global impacts, and as of July 2021, there had been over 185 million confirmed cases of COVID-19 and over 4 million deaths [2]. The virus has at times overwhelmed the health systems of different countries, and the need for accurate and timely information has never been higher. The COVID-19 pandemic has seen users around the world turn to digital technology for the sourcing of health information [3]. Health authorities such as WHO have responded with new and innovative ways of delivering trusted information, including the use of chatbots [4]. Chatbots are software systems that enable users to interact with a program as if they are talking to another person, often using machine learning to achieve the effect of intelligent response. Chatbots are being used across all areas of health to deliver information, promote behavior change, and deliver treatment. Chatbots can be deployed as stand-alone systems or via existing communication platforms such as WhatsApp or Facebook Messenger. This integration into familiar apps can result in a low barrier to entry for many users and a potential for substantial reach.

Chatbots can be rule-based or artificial intelligence–based. Rule-based chatbots use decision trees and defined rules to guide conversations, whereas artificial intelligence–based chatbots uses machine learning and natural language processing to generate and respond to dialogue [5]. As chatbots become more sophisticated, this opens up new and innovative ways to interact with and engage users in digital content. Features such as videos, quizzes, emoji, and style of voice are all used to keep users engaged. As these tools are used more, it is important to understand the marketing and communication strategies used to improve effectiveness and user experience. This enables the design of systems that people want to use and can benefit from.

### Relevant Literature

Good user experience design is important for chatbot adoption and acceptance, and this requires expert input [6]. Digital solutions that are not designed with the user in mind can result in low engagement [7]. Working in a co-design model and involving end users throughout development can help in developing user-centered products and increasing engagement [7]. Chatbots have been developed for a wide range of health issues and behaviors, with mental health chatbots having the most dedicated literature [8]. A recent scoping review of user perspectives and opinions about mental health chatbots found that people generally found them easy to use and that enjoyment and trust are key mediators of interaction with chatbots [9]. However, the quality of conversation was identified as a limitation.

A review of technical metrics used to evaluate chatbots found a diversity in approaches and no apparent standardization [8]. The authors identified 27 metrics related to chatbots as a whole, response generation, response understanding, and aesthetics. They identified a range of ways to measure usability, such as a single question in a questionnaire, multiple questions, observation, or the use of a validated scale such as the System Usability Scale. In addition, they found that only 7% of studies included any assessment of aesthetics [8]. A scoping review of the features most commonly used in mental health chatbots showed that most were rule-based and stand-alone software [10]. In most chatbots, the conversations were led by the chatbot, and most included digital representations such as an avatar or digital human characters [10].

### COVID-19 Chatbots

Chatbots can have special use in a pandemic for reaching people with information, supporting behavior change, providing mental health support, and identifying and monitoring symptoms [11]. Recognizing these opportunities, chatbots for COVID-19 have been quickly developed and scaled up by health authorities such as the WHO [12] and Centers for Disease Control and Prevention [13]. COVID-19 has overwhelmed health systems around the world [14], and the combination of increased need for health services and the need for social distancing has highlighted the potential for chatbots to ease some of the health system burden [15]. A number of studies describe guidance or frameworks for the design of chatbots for COVID-19 [15-17]. Despite the importance of assessing adoption and user engagement, they are not routinely reported in chatbot evaluations, and measures are not standardized [3,4]. The COVID-19 pandemic has presented a unique situation that has accelerated the need for the implementation of digital solutions. Although scoping reviews have previously examined COVID-19 chatbots in general [18], this is the first paper to our knowledge that focuses on this specific scenario. This paper sought to describe the current knowledge base about the user experience of COVID-19 chatbots.

## Methods

### Overview

We conducted a scoping review of the user experience of COVID-19–related chatbots, with regard to design, engagement, and communication features. We followed guidance from the Joanna Briggs Institute on conducting systematic scoping reviews [19] and reported according to the PRISMA (Preferred Reporting Items for Systematic Reviews and Meta-Analyses) guidelines for scoping reviews [20]. This guidance outlines the steps, from the development of a protocol and the search strategy to charting the results and reporting the findings.

### Review Objective

We sought to provide an overview of the evidence of design, engagement, and communication features used in COVID-19 chatbots and the impact on user engagement, preference, and retention.

### Review Questions

Our review questions were as follows:

- What are the best practice approaches to user engagement in COVID-19 chatbots?
- What is known about user experience, preference, and retention in relation to the different engagement strategies, content, and language features of COVID-19 chatbots?
- What marketing and communication strategies have been implemented and evaluated with COVID-19 chatbots?
- What are the gaps in the literature and recommendations for future research?

### Search Strategy

We used a standard 3-step search strategy for scoping reviews.

1. Initial search of MEDLINE and Embase databases followed by an analysis of the keywords and results found
2. Second search using a revised keyword list across all databases
3. Backward and forward citation checking of all the included papers and articles

### Search Sources

For this review, we searched databases from June 19-20, 2021. The search strategy was initially developed for MEDLINE and then adapted for other databases. We initially searched MEDLINE Ovid and Embase. Search results from these databases were reviewed, and the search strategy was adjusted slightly before being rerun. Other databases used in the search included CINAHL, ACM Digital Library, Cochrane COVID-19 study register, Emerald, Communication abstracts (EBSCO), and the WHO COVID-19 Global literature on coronavirus disease. To source unpublished literature, Google Scholar was searched using the adapted search terms and the first 100 results were scanned. Backward and forward citation searches of all the included articles were conducted, and additional articles identified were sourced individually.

### Search Terms

The search terms for this review were first developed with previous knowledge from the authors and then further informed by the current literature about this topic. A comprehensive list of search terms was developed to ensure a broad range of articles. We used search terms related to the technological subject matter of interest (eg, chatbots, conversational agent, and dialogue system), user experience more generally (eg, engagement, features, and user experience), and the health issue at hand (eg, COVID, COVID-19, Corona, and coronavirus). The search terms used for each database can be viewed in Multimedia Appendix 1.

### Inclusion and Exclusion Criteria

We included studies that assessed the design, marketing, and user features of COVID-19 chatbots or those that explored user perspectives on features, marketing, and design. As the aim was to map the existing evidence across published and unpublished literature, there was no criteria limitation with regard to geographical location, type of chatbots, or study design. We included papers, articles, and conference proceedings. Any articles or reports pertaining to chatbots that were not related to COVID-19 were excluded. We excluded papers that did not include any reporting on user perspectives, experience, or the general use of chatbot features or marketing, as well as papers where a version was not available in English. We excluded conference abstracts, books, and theses.

### Study Selection Process

**Figure 1:**
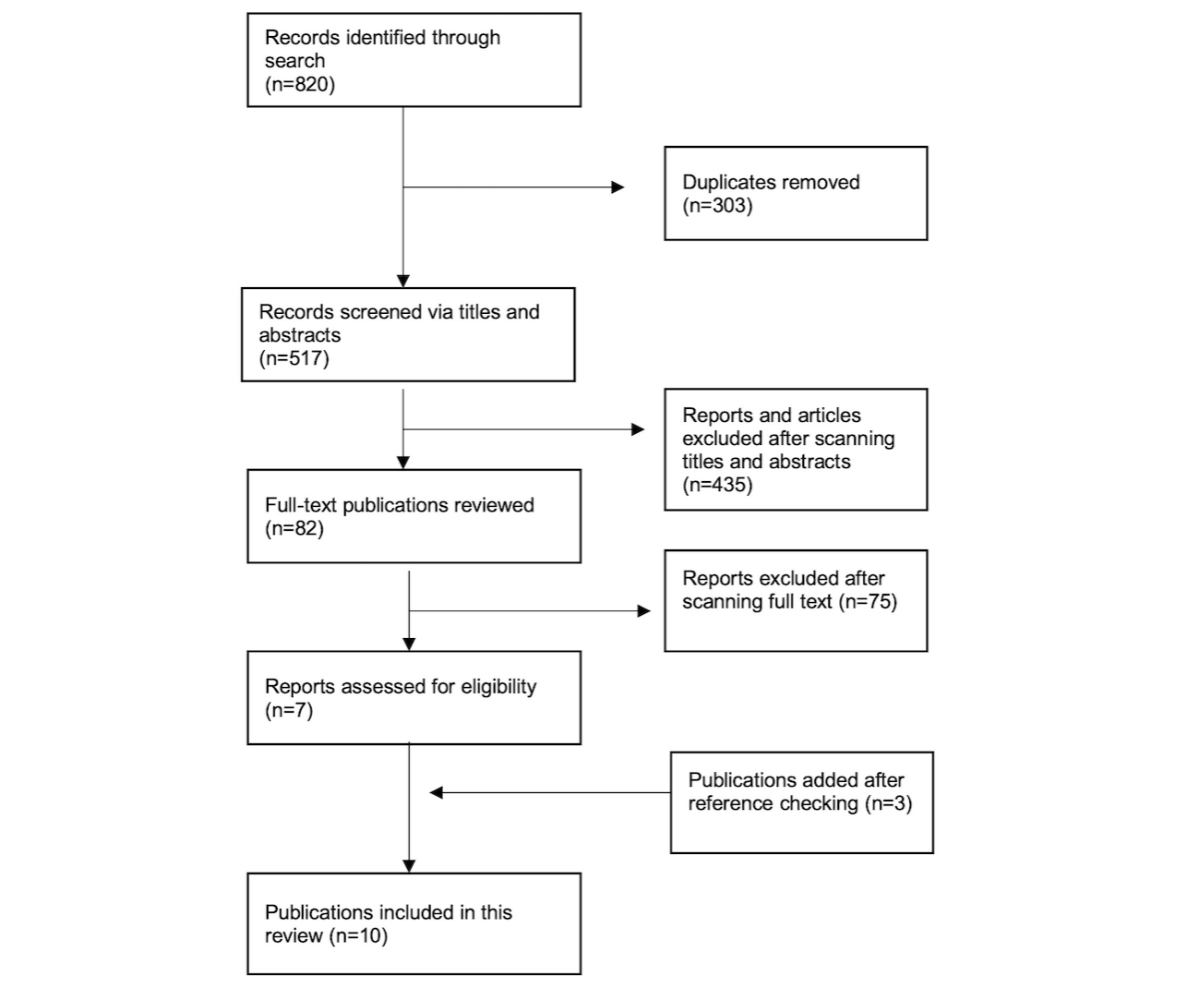
Search strategy and selection process.

The selection of articles was defined by the inclusion and exclusion criteria. In line with the iterative nature of scoping reviews, these criteria were refined as the review progressed with alterations made to the criteria following the initial search of 2 databases. Deduplication was carried out in Endnote software (Clarivate), and then Rayyan was used for screening. Rayyan is a web-based program designed for managing the citation-screening process [21]. Authors BW and AM independently screened article titles and abstracts, excluding irrelevant studies. Any discrepancies were resolved via discussion. For articles deemed possibly relevant or where it was difficult to ascertain from the title and abstract, the full text was reviewed. Figure 1 shows the study selection process.

### Data Extraction and Charting

A data extraction form was developed to ensure the uniform collection of data, and this form was trialed and revised. Data were charted in categories of study characteristics, intervention characteristics, and outcomes. A thematic analysis was then used to synthesize the findings in each paper and identify themes.

## Results

### Included Studies

Of the 517 papers initially identified, 10 were included in the final analysis, with 2 papers reporting results from the same study population. The authors took a wide view of the inclusion criteria, including studies that included any reporting of the abovementioned criteria. The papers differed widely in their approach to reporting user experience, from empirical studies that evaluated user experience comprehensively to those that reported minimal findings. Several papers were excluded even though they had detailed their use experience plan because they did not report findings. Papers included discussions about chatbots developed for COVID-19 screening and general COVID-19 information, as well as studies investigating user perceptions and opinions on COVID-19 chatbots. A thematic analysis was carried out on the included studies to understand and organize the findings across the papers. Table 1 describes the characteristics of the included studies.

**Table 1:** Characteristics of included studies.

Author, year	Chatbot type	Country	Study design	Study aim	Key outcomes
Almalki [22], 2021	Chatbots for COVID-19	Saudi Arabia	Survey, distributed via social media and messaging apps	To explore chatbot use and associated challenges and barriers	40% were aware of chatbots, yet only 24% had used them before 56.9% had positive perceptions 84% expressed willingness to use in future
Almalki [23], 2020	Chatbots for COVID-19	Saudi Arabia	Survey, distributed via social media and messaging apps	To explore user perceptions of health chatbots in Saudi Arabia	Overall positive perceptions Users were more willing to use for general information about COVID-19 Highly educated users were more likely to engage
Dennis et al [24], 2020	COVID-19–screening chatbot	United States	Web-based experiment comparing human or chatbot agents	To understand user response to COVID-19–screening chatbots	User perception of agent ability (human or chatbots) was the primary factor User trust in provider is an important factor
Judson et al [25], 2020	COVID-19–screening chatbot	United States	Descriptive process of development	To describe the development of screening chatbots for a hospital setting	Users need to trust the authority the chatbot is coming from Saved employee time
Liang et al [26], 2021	Chatbots to discuss movies and COVID-19	Not reported	Tested reactions and use with different levels of chatbot disclosure	To understand chatbot self-disclosure, user engagement, and perception	Users’ self-disclosure increased with chatbot’s self-disclosure Chatbots’ self-disclosure also positively impacted engagement and users’ perception
McKillop et al [27], 2021	COVID-19 chatbots using the Watson Assistant platform	Across 9 countries^a^	Descriptive study of the Watson Assistant platform as COVID-19 chatbots	To describe COVID-19 conversational agents built using the Watson Assistant	The average number of conversational turns ranged from 1.9-3.5 Clinical providers had the highest number of turns
Morse et al [28], 2020	COVID-19 symptom checker	United States	Descriptive study of completed assessments	To describe user demographics and levels of triage acuity provided by a symptom checker	30-39 years was most common age group, but a sizable minority were aged 60 years or over Most users were female
Ollier et al [29], 2021	COVID-19 health promotion and health coaching	United Kingdom and Ireland	Descriptive analysis of the marketing of chatbot using Facebook advertisements	To understand the use of Facebook advertisements to drive chatbot use	Static images have better conversions than carousel images Android downloads were higher than iOS Middle-aged older women were more engaged
Rodsawang et al [30], 2020	COVID-19 general information chatbot	Thailand	Descriptive analysis of chatbots development and user feedback	To describe characteristics and user perspectives with the “COVID-19 Preventable” chatbot	Government COVID-19 chatbot with good user feedback and uptake Menu feature became important as users and content increased
Schubel et al [31], 2021	COVID-19 symptom screener and learning module	United States	Survey with people invited to use chatbots over text messaging or email	To understand interaction and feature access by population subgroups	Demographic differences observed, with women, African American individuals, and those aged 51-90 years interacting more

^a^The 9 countries were the United States, Australia, Canada, the United Kingdom, Pakistan, Germany, Russia, Ireland, and Singapore.

### Thematic Analysis

The papers described a range of factors impacting on user experience and chatbot use. The COVID-19 pandemic expedited the development of chatbots with some tools being developed and deployed within 5 days [25,27]. Partnership with technical teams was described as being beneficial [25], with one paper describing the lack of technical expertise on their team as a limitation [30]. Findings are mapped by theme and described in the following section.

#### Factors Impacting User Experience and Use

##### Trust

Trust was explicitly reported in 4 papers [24-26,30]. A user’s perception of the ability of a chatbot was a primary factor impacting interaction according to one paper, and that perception was impacted by the trust placed in the chatbot’s provider [24]. Being clearly marked with the provider’s branding was important, with one study reporting users being suspicious about using a tool that was not clearly branded to their hospital [25]. Users evaluating the “COVID-19 Preventable” chatbot recommended that the user interface should represent public health authorities [30]. As disclosure by one chatbot increased, so did the user’s likelihood of self-disclosure [26]. Increased disclosure by the chatbots also positively affected the engagement and perceived warmth. When the chatbots showed emotional disclosure, engagement significantly increased [26].

##### Digital Literacy

Two papers mentioned digital literacy and ability. Almalki [23] reported that perceived IT skills or past use of chatbots did not impact perceptions of chatbots ability. They found that those who reported frequently searching for health information on the web were more likely to use health chatbots to source medical services [23]. Another paper reported that a user’s perceived digital ability had a small-to-medium effect on satisfaction, motivation, likelihood of use, and adherence to advice [24].

##### Design and Usability

Four papers described the process of design of their chatbots, with a focus on enhancing usability. Two papers described a process of chatbot development that involved stages of defining, design, journey mapping, iteration, and evaluation [25,30]. Another paper examining disclosure with COVID-19 chatbots reported their process of conversation, including starting with small talk and then building up to recommendations [26]. Another paper highlighted the importance of ease of use in maintaining user engagement [30].

##### Demographic Factors

Four studies reported demographics with use and user experience. Schubel et al [31] reported that people aged 51-90 years were the most likely to use their chatbots. However, this differed by feature. Younger users (aged 18-50 years) were more likely to use a symptom screen checker, and older users (years 51-90 years) were more likely to use the learning module. This same study found a greater proportion of African American users than other races or ethnicities and more female users than male users [31].

Conversely, Morse et al [28] reported that although the most common users were aged 30-39 years, there was a sizable minority of older users, with 13.3% being aged 60 years or over. They also found that the majority of completed symptom checker assessments were carried out by female users. Ollier et al [29] reported that female users over the age of 35 years were downloading their chatbots at higher rates than any other group. This was in response to Facebook advertisements promoting the chatbots, and the authors noted that the content of those advertisements may have been more attractive to this group. Almalki [23] reported no significant difference in gender with any of their variables, but that participants aged under 30 years reported more enjoyment with using chatbots [22].

#### User Perspectives on Content and Features

Four papers reported findings related to chatbot content. One paper found that people were more likely to use a chatbot to seek general information about COVID-19 than information about medical treatments [23]. Another paper describing a chatbot with 2 feature components found no difference in the use of the health screener information compared to the general learning content [31]. McKillop et al [27] reported on the average number of conversational turns per session. These ranged from 1.9 to 3.5. Clinician providers had the highest number of turns, suggesting that these chatbots may have been delivering more complex content. The authors suggested that the lower number of conversational turns may be due to people asking more simple question at the start of the pandemic, which may have increased in complexity and, thus, turns as the pandemic continued. Indeed, they saw a marked increase in conversation turns among employees, possibly related to more complex questioning as they looked to return to work [27]. As the number of users increased with one COVID-19 chatbot, so did the range of questions and content, and thus, new systems were needed to manage content [30].

Managing content and daily updates of information during the pandemic was reported as being challenging and constant by the authors describing the “COVID-19 Preventable” chatbot [30]. Identifying user questions that chatbots were unable to answer was a useful way of prioritizing new information and conversation rules to be added. Another challenge was the daily task of translating complex content into deliverable bites for public consumption. Once the use of this chatbot reached over 100,000 users, the use of the menu significantly increased. Content was filtered into categories, and the menu reportedly helped users to navigate and find answers more easily [30]. Progressive disclosure was used to manage information flow from basic to more complex. Additional features were added to this chatbot based on user demand, such as the ability to report a concern about the potential spread of COVID-19 directly to the authorities [30].

#### Marketing

One paper detailed the experience of using Facebook advertisements to promote their chatbot. The authors used A/B testing to test *carousel images* with *static images and text*, finding that *static images with texts* were better received [29]. Android users downloaded and engaged with the chatbots more than iOS users. The authors reported that through their increased engagement with the advertisements, Android users essentially marketed the product to their contacts. Women aged 35 years and over downloaded the chatbots the most. This group also had the highest engagement with the Facebook advertisements themselves, in terms of reacting to and sharing the post [29]. Two other papers, although not reporting specifically on promotion, reported that mass media campaigns, social role model endorsement, or national health authority adoption may help in raising awareness and trust in chatbots [22,23]

#### User Perspectives and Acceptability

The theme of acceptability included willingness to use COVID-19 chatbots, perceptions on chatbots in general, and adoption. Five papers reported some acceptability findings. An employee-screening chatbot deployed at a large hospital site found that adoption was aided by their chatbot not requiring download or a log-in [25]. Despite many of the respondents to one survey having little experience with chatbots, most (82.5%) reported being willing to use chatbots to seek general information about health care services and how to prevent the spread of COVID-19 [23]. Acceptability appeared to be higher for those who already used the internet to seek health information [22]. Participants reported generally having positive perceptions of health chatbots and being willing to use them. Empathy and emotional sensitively was an important factor in acceptability; participants perceived that chatbots had no emotions, which may impact acceptability. Social norms also impact chatbot acceptability, with participants reporting a tendency to align with the views of others [22].

The “COVID-19 Preventable” chatbot reported that most people (98%) stated that they were likely to continue using the chatbot and 96% said that they would recommend it to others [30]. This acceptability may have been aided by the 64% of people who reported that the chatbots answered their questions appropriately [30]. The perceived ability of the chatbot was rated as being important in Dennis et al [24], another study that compared the user reactions of chatbots with human conversations. Interestingly, when ability was perceived to be the same, chatbots were perceived more positively than humans. The authors surmised that this finding may be due to users feeling more comfortable with chatbots when discussing socially challenging information [24].

## Discussion

### Principal Findings

This paper describes what is known about the user experience and user uptake of COVID-19 chatbots. The 10 papers in this review included some describing chatbots for COVID-19 screening, some describing chatbots for information dissemination, and others seeking to understand uptake and user perspectives. The papers generally reported good acceptance of chatbots and reported a number of factors that appeared to determine this. Key themes included content, trust, digital ability, and acceptability. Trust in the chatbot, or in the chatbot provider, was commonly reported as being an important factor. Studies that reported on user acceptability generally found that the chatbots were rated highly, regardless of its type. The number of people seeking health information via digital methods throughout the pandemic has increased [3]; this may have played a role in this acceptability. Digital ability or perceived IT skills were not reported to have a large effect on chatbot use. In some studies, women used the chatbots more than men, and although there were differences in chatbot use or perspectives reported by age, it was clear that chatbots are being used and enjoyed by older people as well as those who are younger. There was no clear indication to whether age and gender play a role in openness to using these tools.

Research presenting results from the United States was overrepresented (5/9, 56% of studies), and the results seen here indicate a need for more diverse reporting and evaluation with COVID-19 chatbots. There have been broader calls in the literature for chatbots responding to COVID-19 to take a global view and be developed with researchers and local data scientists from low- and middle-income countries, to enable better access on a global level [32].

The COVID-19 pandemic has presented challenges to health services and authorities globally, with solutions being developed and rolled out to support countries with accurate and timely health information at a rapid speed. This expediated time frame may have resulted in some of the usual design processes, such as user consultations, message testing, and literature reviews, having not been conducted prior to launch, resulting in phases of evaluation and iteration in real-world settings. The challenge of managing the amount of content in a fast-moving landscape was described by one national government chatbot [30], and partnerships with technical teams were reported as being beneficial [25]. New processes were developed and chatbots have helped to quickly disseminate high-quality information in a time of crisis. These processes and learnings from implementation will be key in informing chatbots for future health emergencies. As well as information delivery, chatbots have the potential to inform information provision and pandemic response by analyzing the questions people are asking or looking at the information they are searching for to identify information voids.

The review revealed a lack of standardized reporting on user experience and user preferences. Although this may be partially explained by the need for expedited deployment, it will be important in the future to revisit and reaffirm best practices in user-focused design and acceptance testing to ensure that these tools are effective. Developing standardized measures for reporting on user experiences with chatbots will help to synthesize the evidence and move it forward in a more cohesive manner. The literature suggests that working in a co-design model involving end users can help with engagement [7], and that usability, enjoyment, and trust are also key factors [9].

It is apparent that further research is needed to better understand user experience, engagement, and uptake, particularly more research from countries outside of the United States. The COVID-19 pandemic has had an unprecedented effect on digital health [33]. Lessons learned on how best to reach people with health information and interventions during the COVID-19 pandemic will have broad application to other health emergencies and future pandemics.

### Limitations

The scoping review summarized the evidence about user experience with COVID-19 chatbots, but as the goal of a scoping review is to map the literature, study quality was not assessed and data synthesis did not occur. The studies that were included reported diverse aspects of user experience, resulting in limitations in drawing together findings on particular components.

### Conclusion

The COVID-19 pandemic presented a unique and specific challenge for digital health interventions in that design and implementation were required at a rapid speed as digital health service adoption was accelerated across the globe. This paper adds to the literature by describing what is known about this rapid implementation process in terms of user experience and user uptake and provides guidance for future tools, as well as directions for future research. This paper has shown both the potential of chatbots to reach users in an emergency and the need to better understand how users engage and what they want.

## Appendix

Multimedia Appendix 1 Search terms.

## Abbreviations

PRISMA: Preferred Reporting Items for Systematic Reviews and Meta-Analyses
WHO: World Health Organization
