# Supplementary material for: User Experience of COVID-19 Chatbots: Scoping Review
Source: J Med Internet Res. 2022 Dec 27;24(12):e35903. doi: 10.2196/35903 (PMC9822175; doi:10.2196/35903)
Supplement: Multimedia Appendix 1 [file jmir_v24i12e35903_app1.docx]

**Appendix 1. Search terms used**

| **Medline and EMBASE** | | |
| --- | --- | --- |
| Concept 1. Chatbots | Concept 2. Engagement features | Concept 3. COVID-19 |
| chatbot*  "conversational agent*"  “conversational system*”  "relational agent*"  "dialogue system*"  "Apple Siri"  "Amazon Alexa"  "Cortana"  "Google Home"  "Google Assistant"  Whatsapp  Viber  “Facebook messeng*”  chatterbot*  “chat bot*”  chat-bot*  “Agent-based system”  Smart-bot*  “Smart bot*”  “Dialog system*” | engag*  “user experience”  "content”  Motiv*  usage  “sticker*”  quiz*  language  menu*  feature*  Emoji  Readability  Game*  video*  flash card*  locali?ation  promotion  marketing  “user-centric”  “human-centric”  UX  “conversational UX”  “UI design”  UI  ad*  advertisement* | COVID  COVID-19  Corona  Coronavirus |

| **Database** | **Search terms** |
| --- | --- |
| Emerald Search | (COVID-19 OR COVID OR Corona OR Coronavirus) AND (Chatbot OR "conversational agent" OR “conversational system*” OR "relational agent*" OR "dialogue system*" OR "Apple Siri"OR "Amazon Alexa" OR "Cortana" OR "Google Home" OR "Google Assistant" OR“Whatsapp” OR “Viber” OR “Facebook messeng*” OR ”chatterbot*” OR chat bot* OR chat-bot* OR “Agent-based system” OR Smart-bot* OR “Smart bot*” OR “Dialog system*”) |
| CINAHL | (“chatbot*" or "conversational agent*" or “conversational system*” or "relational agent*" or "dialogue system*" or "Apple Siri" or "Amazon Alexa" or "Cortana" or "Google Home" or "Google Assistant" or “Whatsapp” or “Viber” or “Facebook messeng*” or ”chatterbot*” or chat bot* or chat-bot* or “Agent-based system” or Smart-bot* or “Smart bot*” or “Dialog system*”) AND (“engag*” or “user experience” or "content” or Motiv* or usage or “sticker*” or “quiz*” or “language” or “menu*” or “feature*” or “Emoji” or “Readability” or “Game*” or “video*” or“flash card*” or “locali?ation” or “promotion” or “marketing” or “user-centric” or “human-centric” or “UX” or “conversational UX” or “UI design” or “UI” or “ad*” or “advertisement*”) AND (COVID OR COVID-19 OR Corona OR Coronavirus) |
| ACM digital library | (“chatbot*" or "conversational agent*" or “conversational system*” or "relational agent*" or "dialogue system*" or "Apple Siri" or "Amazon Alexa" or "Cortana" or "Google Home" or "Google Assistant" or “Whatsapp” or “Viber” or “Facebook messeng*” or ”chatterbot*” or chat bot* or chat-bot* or “Agent-based system” or Smart-bot* or “Smart bot*” or “Dialog system*”) AND (“engag*” or “user experience” or "content” or Motiv* or usage or “sticker*” or “quiz*” or “language” or “menu*” or “feature*” or “Emoji” or “Readability” or “Game*” or “video*” or“flash card*” or “locali?ation” or “promotion” or “marketing” or “user-centric” or “human-centric” or “UX” or “conversational UX” or “UI design” or “UI” or “ad*” or “advertisement*”) AND (COVID OR COVID-19 OR Corona OR Coronavirus) |
| Cochrane COVID-19 study register | (chatbot OR “conversational agent” OR “relational agent” OR “dialogue system” OR “Apple Siri” OR “Google Alexa” OR Cortana OR “Google Home” OR “Google Assistant” OR Whatsapp OR Viber OR “Facebook messenger” OR chatterbot, OR “conversational system” OR “chat bot” OR chat-bot) AND  (content OR design OR stickers OR quizzes OR language OR “user experience” OR menus OR engagement OR features OR Emoji OR Readability OR Games OR video OR “flash cards” OR locali?ation OR promotion OR marketing OR user-centric OR human-centric OR UX OR “conversational UX” OR UI design OR UI OR ads OR advertisements) |
| WHO COVID-19 Global literature on coronavirus disease | (chatbot OR conversational agent OR relational agent OR dialogue system OR apple siri OR google alexa OR cortana OR google home OR google assistant OR whatsapp OR viber OR facebook messenger OR chatterbot, conversational system OR chat bot OR chat-bot) AND (content OR design OR stickers OR quizzes OR language OR user experience OR menus OR engagement OR features OR emoji OR readability OR games OR video OR flash cards OR locali?ation OR promotion OR marketing OR user-centric OR human-centric OR ux OR conversational ux OR ui design OR ui OR ads OR advertisements) |
| Google scholar search | (“Chatbot*” OR “Whatsapp” OR “conversational agent”) AND (engag* OR feature* OR “Content” OR “promotion” OR “video” OR “game”) AND (“COVID-19” OR “COVID*” OR “Corona*” OR “Cornonavirus*”) |
